# Adipose Tissue-Secreted Factors Alter Bladder Cancer Cell Migration

**DOI:** 10.1155/2018/9247864

**Published:** 2018-05-20

**Authors:** Nisha Hariharan, Keith A. Ashcraft, Robert S. Svatek, Carolina B. Livi, Desiree Wilson, Dharam Kaushik, Robin J. Leach, Teresa L. Johnson-Pais

**Affiliations:** ^1^Department of Cell Systems and Anatomy, University of Texas Health Science Center, San Antonio, TX 78229, USA; ^2^Department of Urology, University of Texas Health Science Center, San Antonio, TX 78229, USA

## Abstract

**Background:**

Obesity is associated with an increased risk of bladder cancer recurrence. This study investigated the role of adipose tissue in bladder cancer progression.

**Methods:**

Gene expression profiling was performed on adipose tissues collected from normal weight (*n*=5), overweight (*n*=11), and obese (*n*=10) patients with invasive bladder cancer, and adipose stromal cells (ASCs) were obtained from two normal weight, two overweight, and two obese patients. Conditioned media (CM) was characterized and evaluated for its effects on the proliferation, migration, and invasive potential of T24 bladder cancer cells.

**Results:**

Expression profiling demonstrated depot-specific or body mass index-specific differences. Increased T24 cell migration was observed using CM harvested from all ASCs. ASC CM from an obese patient significantly increased T24 cell migration and invasion compared to ASC CM collected from normal weight and overweight patients. We identified abundant expression of CXCL1, PAI1, IL6, CX3CL1, and CCL2 in all CM. Exogenous treatment of T24 cells with PAI1, IL6, and CXCL1 enhanced migration. Depletion of CXCL1, PAI1, and IL6 in an obese patient ASC CM abrogated T24 migration.

**Conclusion:**

Factors secreted by adipose tissue influence the migration of bladder tumor cells and could play an active role in tumor progression.

## 1. Introduction

An estimated 79,000 urinary bladder cancer cases will be newly diagnosed and are expected to result in 16,870 deaths in the United States in 2017 [1]. Bladder cancer is the fourth most common cancer among American men and the eighth most common cause of cancer deaths [1, 2]. The bladder is surrounded by visceral adipose tissue, and bladder cancer staging is based on involvement of perivesical fat. Tumors with extravesical invasion of the tumor into the perivesical fat (pathologic stage T3) are highly predisposed to lymph node involvement and distant metastasis [3, 4]. Obesity appears to play an important role in the development of bladder cancer. An epidemiological study of 1,719 incident bladder cancer cases demonstrated that obese individuals had a 28% increased risk for bladder cancer compared to normal weight individuals [5]. Obesity has been shown to be associated with worse cancer-specific outcomes in bladder cancer patients in a large multi-institutional cohort. In patients with body mass index (BMI) > 30 kg/m^2^, there was a 67% increased risk of cancer recurrence and a 43% increased risk of cancer-specific mortality [6]. In addition, a meta-analysis of 14 prospective cohort studies showed a positive association between bladder cancer and obesity and a potential association between BMI and bladder cancer risk with the risk increasing by 3.1% for each 5 kg/m^2^ increase [7].

Several studies have reported a positive association of obesity severity with adverse outcomes in many cancer types [8–10]. Studies of obesity and prostate cancer revealed that the periprostatic adipose tissue secretes high levels of interleukin-6 (IL6), compared to subcutaneous adipose tissue, which modulates cancer aggressiveness [11, 12]. Subcutaneous and visceral adipose depots show differential patterns of gene expression in obese patients with and without endometrial cancer [13]. Adipose tissue is heterogeneous, and the stromal vascular fraction that is enriched for the adipose stromal cells (ASCs) is a potent source of numerous cytokines and chemokines [14]. The altered expression of the various secreted factors from the adipose tissue in the overweight and obese states has been observed to have mitogenic effects in numerous cancers [15–21].

Here, we sought to gain an understanding of the role of adipose tissue in bladder cancer and to determine whether bladder tumors are affected by the dysfunctional adipose tissue in the obese state. We characterized the gene expression profiles of adipose tissue collected from various patients across a spectrum of BMI. In addition, we observed that secreted factors from bladder cancer patients' adipose tissue influence the proliferation and migratory and invasive capacity of human bladder cancer cells.

## 2. Materials and Methods

### 2.1. Patient Recruitment and Tissue Procurement

All adipose tissue samples were obtained through a bladder cancer repository maintained in the Department of Urology that has full institutional review board (IRB) approval at the University of Texas Health Science Center at San Antonio. Fresh adipose tissue samples were provided without identifiers The research protocol for this study was assessed by the IRB and determined to be nonhuman research (protocol number: HSC20130350N). Adipose tissue was obtained from 26 male patients who were undergoing radical cystectomy for locally advanced bladder cancer. Adipose tissue was collected from the fat surrounding the urinary bladder and subcutaneous tissue. Care was taken to extract fat surrounding the bladder and when tumor was present, immediately adjacent to the tumor site. This method was the same for all patients/samples. When no bladder tumor was present, fat was removed from the scarred area (resection site) which corresponded to where the tumor was before transurethral resection. An assessment for the presence of contaminating tumor cells in the whole fat surrounding the bladder was not performed. The patients were stratified into three BMI groups according to the World Health Organization standards: normal weight (*n*=5, BMI < 25 kg/m^2^), overweight (*n*=11, BMI = 25–29.9 kg/m^2^), and obese (*n*=10, BMI ≥ 30 kg/m^2^).

### 2.2. Adipose Tissue Explant Conditioned Media

Specimens (0.5–2.0 g) of the bladder and subcutaneous adipose tissue, when available, and based on the quantity of available tissues were placed in primary whole adipose tissue explant cultures using a modified protocol adapted from Thalmann et al. [22]. Adipose tissue (500 mg) was incubated as an explant overnight in the serum-free Dulbecco's modified Eagle's–Ham's F-12 medium (DMEM/F12) (1 : 1 v/v) (Corning, Manassas, VA) supplemented with 1% penicillin-streptomycin and antimycotic agent. The explant cultures were maintained at 37°C in a 5% CO_2_ atmosphere for 48 hours. The explant culture media was subsequently collected, centrifuged, and frozen at −80°C as whole adipose tissue explant conditioned media (FCM).

### 2.3. Isolation of Adipose Stromal Cells (ASCs) and Preparation of ASC CM

Bladder and subcutaneous adipose tissue (1-2 g) from normal weight (*n*=2), overweight (*n*=2), and obese patients (*n*=2), from whom there was sufficient tissue available, was digested with Blendzyme (Roche, Indianapolis, IN) to release the individual cell components, placed in a shaker rotating at 75 rpm for 60 minutes, filtered with 100-micron mesh, and plated on 60 mm tissue culture dishes [23]. The ASCs were expanded in DMEM/F12 with 10% fetal bovine serum (FBS). To prepare ASC conditioned media (ASC CM), 7.5 × 10^4^ ASCs seeded in DMEM/F12 media supplemented with 2% FBS and 1% penicillin and amphotericin-B in 35 mm dishes were allowed to adhere and then washed with phosphate-buffered saline (PBS) and switched to serum-free DMEM/F12 media supplemented with 1% penicillin and amphotericin-B. After 48 hours, ASC CM was collected and stored at −80°C.

### 2.4. Morphology and Flow Cytometry Analysis of Patient-Derived ASCs

ASC cultures from early passages (1–6 population doublings) were harvested and resuspended in DMEM/F12 with 2% FBS. The cells were then centrifuged and suspended in cold PBS at a concentration of 10^6^ cells/100 *μ*l. Cell aliquots were stained with monoclonal mouse anti-human antibodies against the following antigens: CD44-peridinin-chlorophyll-protein/cyanin 5.5, CD90-phycoerthyrin (PE), PE/Cy7-CD105, CD73-fluorescein isothiocyanate, CD34-Alexa Fluor 647, CD45-Pacific Blue (PB), CD11b-PB, and CD31-PB (BioLegend, San Diego, CA) for 30 minutes in the absence of light at 4°C. The cells that were stained with a single antibody coupled with a fluorescent dye were acquired for compensation purposes. After incubation, the cells were washed and resuspended in cold PBS. Flow cytometry was performed using a Becton Dickinson LSR II. A gate was set to include only the viable propidium iodide-negative cells. The number of cells staining positive for a given cell surface marker was determined by the percentage of cells present within an established gate. A minimum of 10,000 events were counted for each analysis.

### 2.5. Adipogenic Differentiation of Patient ASCs

Adipogenic differentiation of ASCs was performed as previously described [24]. Subcutaneous and bladder-associated ASCs plated in the growth media were cultured until confluence in a 35 mm dish. The medium was removed, and the cells were incubated with or without the adipogenic differentiation medium consisting of the DMEM/F12 medium supplemented with 10% FBS with the addition of 1 *µ*M dexamethasone (Sigma-Aldrich, St. Louis, MO), 10 *µ*g/ml recombinant human insulin, and 0.5 mM 1-methyl-3-isobutyl xanthine (IBMX) (Sigma-Aldrich) for three days. This medium was replaced every three days with the same medium without IBMX for the adipogenic differentiation maintenance period. After 15 days of differentiation, stromal and adipogenic cells were fixed in 1% paraformaldehyde in PBS and stained using the lipid stain Oil Red O.

### 2.6. Gene Expression

Total RNA was isolated from adipose specimens of approximately 100 mg in weight. The frozen patient adipose tissue was homogenized in the QIAzol lysis reagent (Qiagen, Valencia, CA), and the ASCs were disrupted using a Mini-Beadbeater-16 cell disrupter (BioSpec, Bartlesville, OK). Once homogenized, chloroform was added and the homogenate was centrifuged to separate the aqueous phase from the organic phase. The aqueous phase, containing the RNA, was transferred to a new tube and processed with the RNeasy lipid tissue mini kit (Qiagen) for the whole adipose tissue and RNeasy mini kit (Qiagen) for the ASCs, according to the manufacturer's instructions. RNA quantity was assessed using a NanoDrop spectrophotometer (Thermo Fisher Scientific, Waltham, MA), and RNA integrity and purity were assessed using the Agilent 2100 Bioanalyzer (Agilent Technologies, Santa Clara, CA). For cRNA probe labeling, 500 ng of total RNA was used in the Illumina TotalPrep RNA Amplification protocol to create the cRNA probe (Thermo Fisher Scientific, Waltham, MA). The hybridization cocktail containing the labeled cRNA probes was hybridized to the Human HT-12v4 Whole-Genome Expression BeadChip microarray (Illumina, San Diego, CA). The arrays were subsequently processed and imaged using the Illumina iScan, according to the manufacturer's protocol. GenomeStudio software (Illumina) was used for background correction, data normalization, and filtering. Data analysis was performed using GeneSpring 12.6 (Agilent Technologies). Gene function and network enrichments for selected genes were performed using Pathway Architect. The threshold set for significantly dysregulated genes was a *p* value < 0.05 and a twofold or higher change. The moderated *T*-test was used to compare gene expression changes between groups, and a *p* < 0.05 was considered statistically significant. A candidate gene prioritization analysis was performed on the identified gene transcripts based on functional annotations using the ToppGene Suite [25].

### 2.7. Measurement of Protein Levels

Potential protumor factors in the conditioned medium obtained from whole fat explants (FCM) from different anatomical sites from six of the 26 patients for whom there was sufficient tissue available (normal, *n*=1; overweight, *n*=2; obese, *n*=3) and from patient ASC cultures (ASC CM) (normal, *n*=2; overweight, *n*=2; obese, *n*=2) were screened for 41 secreted cytokines/growth factors and six adipokines using the MILLIPLEX MAP 41-plex Human Cytokine/Chemokine Panel and the Human Adipokine Panel 1 (Billerica, MA), which were evaluated using FLEXMAP 3D™ (Luminex, Austin, TX).

### 2.8. Cell Lines and Culture Conditions

The human transitional cell carcinoma cell line T24 was purchased from ATCC and cultured as recommended in McCoy's 5A media supplemented with 10% FBS, 1% penicillin-streptomycin, and antimycotic agent. The cell line was authenticated using short tandem repeat DNA fingerprinting by the University of Texas MD Anderson Cancer Center Characterized Cell Line Core, NCI # CA016672. Cell viability was determined using trypan blue dye exclusion.

### 2.9. Cell Proliferation

Approximately 2 × 10^3^ T24 cells were plated in triplicate in a 96-well flat-bottomed cell culture plate, and each well was treated with (1) whole fat conditioned media (FCM), (2) media only (negative control), or (3) ASC CM. After 24 hours in culture, cell proliferation was assessed using the CellTiter 96 AQ MTS (3-(4,5-dimethylthiazol-2-yl)-5-(3-carboxymethoxyphenyl)-2-(4-sulfophenyl)-2H-tetrazolium) assay (Thermo Fisher Scientific) following the manufacturer's protocol.

### 2.10. Cell Migration Using the Transwell System

T24 cells (2 × 10^5^ cells) were plated in duplicate on the top of 8-micron Transwell inserts (BD Biosciences, San Jose, CA) and exposed to serum-free media (SFM) (negative control), whole fat explant FCM (diluted 1 : 6 in SFM), or ASC CM (diluted 1 : 6 in SFM) in the lower chamber. FCM and ASC CM were diluted 1 : 6 to demonstrate that the effects were not nonspecific effects of high levels of protein present in the CM. The positive control consisted of the complete culture medium as the chemoattractant. The cells were allowed to transmigrate across the porous membrane at 37°C in a 5% CO_2_ incubator. After the cells were incubated for 18 hours, the inserts were removed, and tumor cells on the upper membrane were detached with a sterile cotton swab. Inserts were stained with the Hema-3 staining system (Thermo Fisher Scientific). Pictures of the migrated cells were taken in five random microscopic fields per insert with 150x magnification using a Nikon Eclipse Ti bright-field microscope, and the average number of migrated cells/fields was determined by counting.

### 2.11. Cell Invasion Assay Using Matrigel

Cell invasion was assayed using a reconstituted extracellular matrix membrane (Matrigel; BD Biosciences, San Jose, CA). T24 cells (1.8 × 10^5^ cells) were seeded in duplicate in the upper well of Transwell chambers precoated with 20 *μ*g Matrigel. The media tested in the lower chamber was (1) explant CM (diluted 1 : 6 in SFM), (2) ASC CM (diluted 1 : 6 in SFM), or (3) SFM (negative control). The positive control included the complete culture medium in the lower well. The cells were incubated for 21 hours at 37°C in a 5% CO_2_ incubator. Following incubation, noninvading cells on the top of the upper chamber were removed using a sterile cotton swab, and the cells that invaded through the Matrigel were stained with the Hema-3 staining system (Fisher Scientific). The invading cells were counted in five random microscopic fields per insert with 150x magnification using a Nikon Eclipse Ti bright-field microscope.

### 2.12. Stimulatory Effect of Selected Secreted Factors on Bladder Cancer Cell Migration

To test whether the cytokines identified in the conditioned media mediated the bladder cancer cell migration, media containing the following selected recombinant factors: interleukin-8 (IL8) at 10 ng/ml, chemokine (C-X-C motif) ligand 1 (CXCL1) at 10 ng/ml, plasminogen activator inhibitor 1 (PAI1) at 40 nM, interleukin-6 (IL6) at 5 ng/ml, fractalkine (CX3CL1) at 10 ng/ml, and monocyte chemoattractant protein 1 (CCL2) at 1 ng/ml, was placed in the lower well of a Transwell system. The concentration of the factors was determined from the quantities detected in the CM. T24 cells (2 × 10^5^ cells) were plated in duplicate on the top of 8-micron Transwell inserts, and cell migration was assayed as described above. In addition, the activity of the IL6, PAI1, and CXCL1 was also individually blocked by the addition of the respective neutralizing antibody in the lower chamber. The IL6 and CXCL1 antibodies were obtained from R&D Systems (Minneapolis, MN), and the PAI1 antibody was obtained from EMD Millipore (Billerica, MA).

### 2.13. Depletion of Secreted Factors in Patient ASC CM

ASC CM from an obese patient was depleted of IL6, PAI1, and CXCL1 by incubation with neutralizing antibodies against the three factors (described above) at 4°C overnight with constant rotation. The antibodies were removed by adding 20 *μ*l/ml Protein-G agarose beads (GE Healthcare Life Sciences, Pittsburg, PA) and incubating for four hours with rotation. CM containing the agarose beads was spun at 260 ×g for three minutes, and the supernatant was collected for use in the migration assay [26]. The positive control ASC CM was not incubated with the neutralizing antibodies but were treated with the Protein-G agarose beads. T24 cells (1.8 × 10^5^ cells) were plated in duplicate on the top of 8-micron Transwell inserts (BD Biosciences), and the CM (depleted and control) was added in the lower wells. The cells were allowed to migrate for 21 hours. The Transwell inserts (BD Biosciences) were subsequently stained with the Hema-3 staining system to detect the migrated cells (Thermo Fisher Scientific). The migrated cells were counted in five random microscopic fields per insert with 150x magnification using a Nikon Eclipse Ti bright-field microscope.

### 2.14. Statistical Analysis

One-way ANOVA tests followed by multiple comparison testings were performed using the GraphPad Prism software, version 5.00 (GraphPad Software, San Diego, CA; www.graphpad.com) to determine statistically significant differences between groups. Differences were considered significant if *p* ≤ 0.05.

## 3. Results

A total of 26 male bladder cancer patients who had invasive bladder cancer provided adipose tissue for analysis (patient demographics in Supplementary Table 1 in Supplementary Material available online). The patients were between 56 and 85 years of age. Approximately 70% of the patients were current or former smokers, 31% of them had previously received bacillus Calmette–Guérin (BCG) treatment, and 31% of them received treatment with neoadjuvant chemotherapy. At the time of cystectomy, downstaging of the tumor was observed in most of the patients and is a common occurrence in patients who receive neoadjuvant chemotherapy. Thus, the patients can have less than T2 disease at cystectomy, including T0-T1. The patient BMI range for each category was normal (19.21–24.86), overweight (25.07–29.97), and obese (30.08–41.91) Results were not stratified based on smoking, BCG therapy, or chemotherapy treatment.

**Figure 1:**
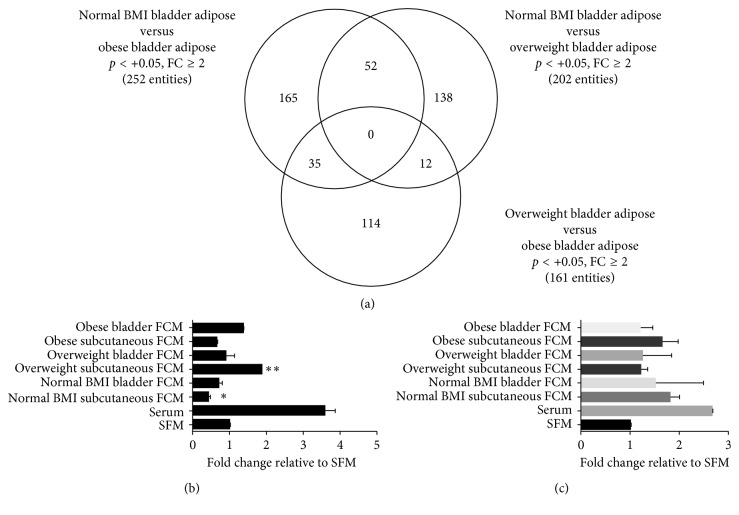
Gene expression profiling of bladder adipose tissue and effects of the influence of explant CM on T24 cancer cell behavior. (a) A Venn diagram representative of gene transcripts differentially regulated in the patient bladder fat between the groups: normal BMI versus obese, normal BMI versus overweight, and overweight versus obese. (b) Adipose whole explant CM from a normal weight patient, an overweight patient, and an obese patient was placed in the bottom of a Boyden chamber, and T24 cells were placed in the upper chamber. Serum-free media (SFM) was used as a control. Migrated cells were stained and counted. ^∗^ *p* < 0.05  and  ^∗∗^ *p* < 0.01 are statistically significant. (c) Fat CM harvested from a normal weight patient, an overweight patient, and an obese bladder cancer patient was placed in the bottom of a Transwell invasion chamber, and T24 cells were placed in the upper chamber. Serum-free media (SFM) was used as a control. Invasive cells were stained and counted. ^∗^ *p* < 0.05  and  ^∗∗^ *p* < 0.01 are statistically significant.

Whole-genome gene expression was performed using RNA isolated from visceral adipose tissue surrounding the bladder from normal weight (*n*=5), overweight (*n*=11), and obese (*n*=10) men with bladder cancer. A summary of the relationship between the differentially expressed genes in adipose tissue is presented as a Venn diagram in Figure 1(a). The analysis demonstrated that when comparing the gene expression profiles from obese versus normal weight men, 252 gene transcripts were found to be differentially expressed (*p* < 0.05 at a minimum of twofold differential expression). A candidate gene prioritization analysis was performed on the 252 gene transcripts based on functional annotations using the ToppGene Suite [25]. The top biological process in which these genes are involved in is secretion, with a total of 28 genes or 11% of the genes. Two hundred and two significant gene transcripts were found to be at least twofold differentially regulated (*p* < 0.05) when comparing the gene expression patterns between the adipose tissue surrounding the bladder of normal weight men and overweight men. A candidate gene prioritization performed on these 202 gene transcripts based on functional annotations using the ToppGene Suite revealed immune response, defense response, inflammatory response, regulation of the immune system process, and leukocyte activation as the top biological processes. Interestingly, the molecular functional annotation identified that the cytokine-cytokine receptor interaction pathway involved 12 of the differentially expressed genes. Comparison of gene expression patterns of bladder adipose tissue between obese and overweight men identified 161 genes that were at least twofold differentially regulated (*p* < 0.05). Candidate gene prioritization of the 161 genes using ToppGene revealed regulation of secretion, locomotion, regulation of the apoptotic process, regulation of the programmed cell death, and regulation of secretion by cells as the top biological processes.

To understand how adipose tissue contributes to paracrine signaling in bladder cancer, FCM from whole fat explants was assayed for their ability to affect the migration and invasion potential of T24 human bladder cancer cells. FCM from matched bladder and subcutaneous adipose tissue from three patients had a variable effect on T24 migration (Figure 1(b)). FCM also did not stimulate the invasive capability of T24 cells (Figure 1(c)). Since we observed a large variability in the migration of T24 cells when exposed to FCM across the different specimens, and because adipose tissue is a mixture of different cell types with the possibility that contaminating tumor cells may have been present in the whole fat, we chose to purify and expand the ASC population from the whole bladder fat and subcutaneous fat for further analysis.

**Figure 2:**
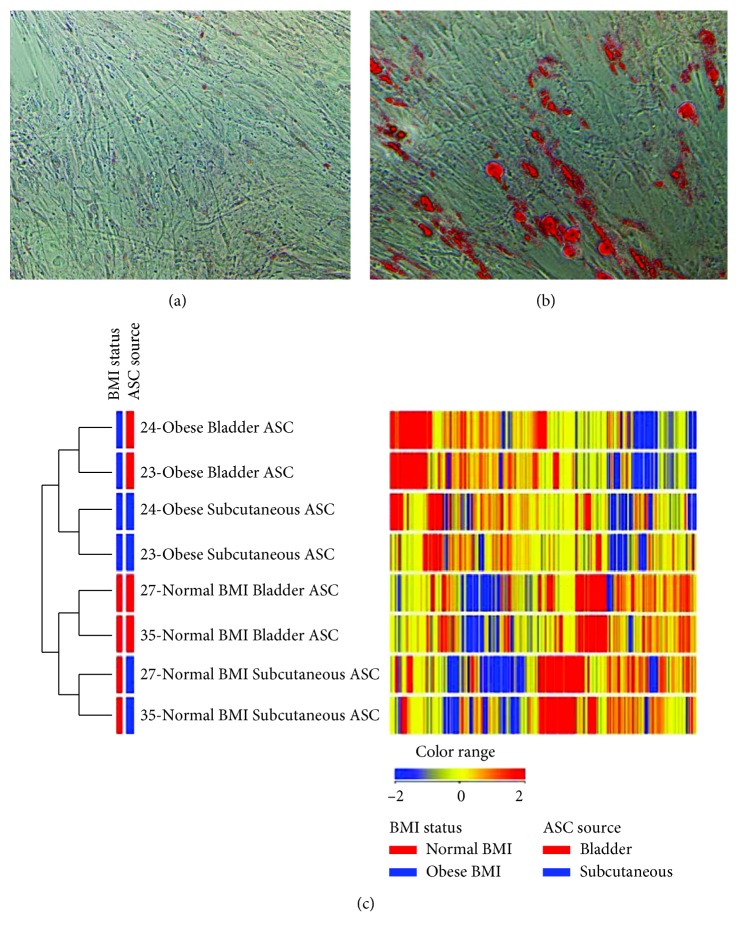
Characterization of bladder ASCs. Primary ASC cultures were allowed to grow to confluence in 6-well plates and were treated (a) without or (b) with the adipogenic differentiation medium. Adipogenic cells were stained using Oil Red O. (c) A heat map representative of differential gene expression in ASCs isolated from matched bladder and subcutaneous sites from two normal weight and two obese patients shows clustering based on the BMI status and ASC source. Transcripts are at least twofold differentially regulated (*p* < 0.05). The heat map indicates upregulation (red), downregulation (blue), and comparable levels of gene expression (yellow) and clustering based on the anatomical source of ASCs, as well as the BMI. PT = patient ID; BL = bladder; SQ = subcutaneous.

To characterize the cells isolated as ASCs, selected ASC cultures were analyzed for the expression of common stem cell surface markers using flow cytometry. The subcutaneous and bladder ASCs tested positive for the cell surface markers CD73, CD44, CD90, CD105, and CD34 and negative for CD45, CD11b, and CD31 (data not shown). The cultured ASCs show similar cell surface markers as those of mesenchymal stem cells. Furthermore, ASC cultures treated with adipogenic differentiation media were able to undergo adipogenic differentiation, which was demonstrated by the staining of fat droplets using Oil Red O (Figures 2(a) and 2(b)). Gene expression analysis was performed using ASCs isolated from matched bladder and subcutaneous adipose tissue from two normal weight and two obese patients. The gene expression patterns of the ASC cluster based on both fat site and BMI status (Figure 2(c)). Bladder ASCs have a unique expression signature compared to subcutaneous ASCs. Comparison of gene expression profiles between ASCs derived from the subcutaneous and bladder depots in an obese patient revealed 15,394 differentially expressed transcripts with 7,615 transcripts that were at least twofold upregulated in the bladder depot showing a stratification based on BMI as well.

**Figure 3:**
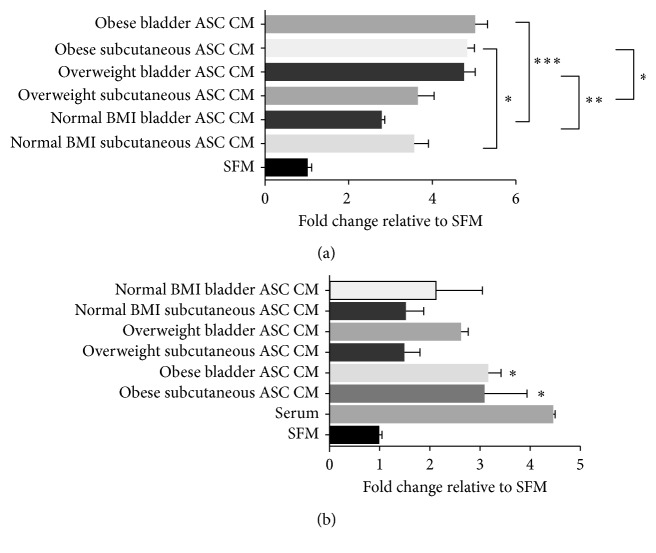
Effect of ASC CM on the migration and invasion potential of T24 cells. (a) ASC CM harvested from a normal weight patient, an overweight patient, and an obese bladder cancer patient was placed in the bottom of a Transwell invasion chamber, and T24 cells were placed in the upper chamber. Migrated cells were stained and counted. ^∗^ *p* < 0.05, ^∗∗^ *p* < 0.01, and  ^∗∗∗^ *p* < 0.001 are statistically significant. (b) ASC CM harvested from a normal weight patient, an overweight patient, and an obese bladder cancer patient was placed in the bottom of a Transwell Matrigel invasion chamber, and T24 cells were placed in the upper chamber. Invasive cells were stained and counted. ^∗^ *p* < 0.05 is statistically significant.

Conditioned media was prepared from ASC cultures derived from bladder and subcutaneous adipose tissue from a normal weight patient, an overweight patient, and an obese patient, and this media was able to induce migration of T24 cells (Figure 3(a)). Also, the ASC CM showed a greater effect in inducing T24 cell migration than what was previously observed with the explant CM (Figure 1(b)). Interestingly, the ASC CM (from both subcutaneous and bladder adipose tissue) obtained from an obese patient was able to cause a significant increase in T24 cell migration, compared to the respective depot-specific ASC CM obtained from a normal weight patient (Figure 3(a)). These results suggest that adipose stromal cells secrete factors that enhance the ability of T24 cells to migrate. Since it is difficult to control for the concentration of these factors in the explant FCM between specimens due to differences in the cellularity of the specimens, this may explain the variability in the stimulation of migration between the explant FCM and ASC CM. Only ASC CM obtained from an obese patient was also able to cause a significant increase in the invasiveness of T24 cells (Figure 3(b)). Conditioned media from whole fat or ASCs did not influence the proliferation rate of T24 cells *in vitro* (data not shown).

To determine which secreted factors may be influencing T24 behavior, the whole adipose tissue explant FCM and ASC CM were evaluated for the presence of 41 known cytokines/growth factors and six adipokines. In the adipose CM, we found that interleukin-8 (IL8), interleukin-6 (IL6), GRO1 oncogene (CXCL1), and fractalkine (CX3CL1) were present in high levels among several others (total 25 factors) detected above control SFM. The adipokine plasminogen activator inhibitor 1 (PAI1) was also present in high levels. High levels of monocyte chemoattractant protein 1 (CCL2), CXCL1, IL6, IL8, and PAI1 were also found in patient ASC CM, irrespective of patient BMI. We chose to focus our investigations on the secreted factors that were detected in both the adipose CM and ASC CM. Data from studies of the bladder and other cancers implicate IL6, IL8, CXCL1, and PAI1 as factors that may play a role in the ability of the adipose CM and ASC CM to alter the migratory potential of the T24 cells [18, 27–32].

**Figure 4:**
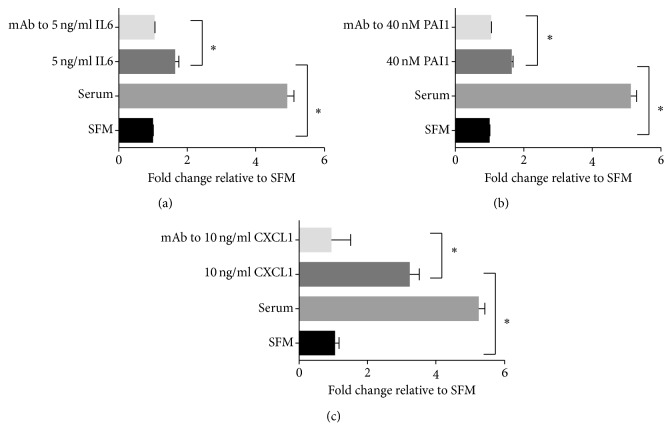
The activities of IL6, PAI1, and CXCL1 stimulate T24 migration. (a) Recombinant IL6 was added to serum-free media placed in the bottom of a Transwell chamber. Neutralizing antibody (Ab) against IL6 was added to block the activity. (b) Recombinant PAI1 was added to serum-free media placed in the bottom of a Transwell chamber. Neutralizing Ab against PAI1 was added to block the activity of PAI1. (c) Recombinant CXCL1 was added to serum-free media placed in the bottom of a Transwell chamber. Neutralizing Ab against CXCL1 was added to neutralize the CXCL1 activity. To each treatment, serum-free media (SFM) was used as a control. Migrated cells were stained and counted. ^∗^ *p* < 0.05 is statistically significant.

**Figure 5:**
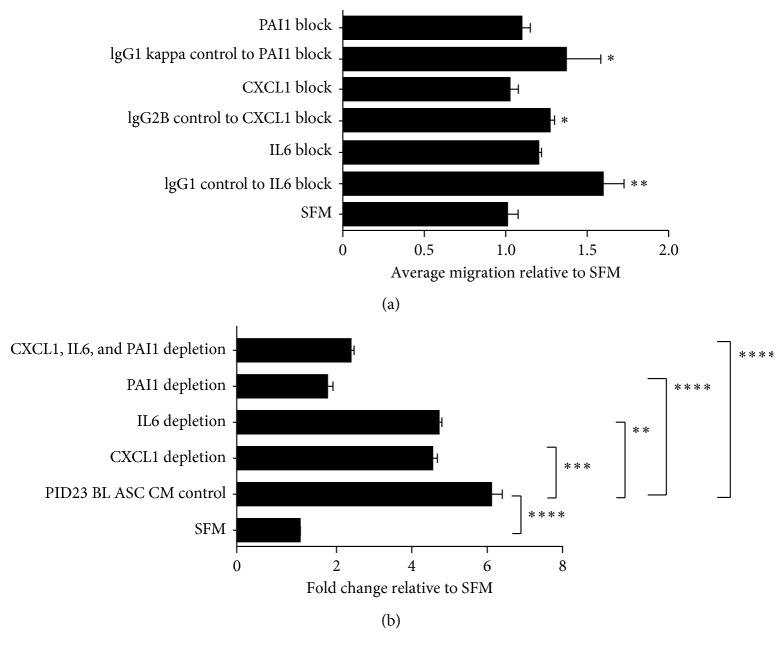
Blocking or depleting IL6, CXCL1, and PAI1 reduces migration of T24 cells. (a) Blocking the activity of exogenously added recombinant IL6, CXCL1, and PAI1 with neutralizing antibodies significantly reduces T24 migration in a Transwell assay compared to treatment with an isomeric IgG control. One-way ANOVA with uncorrected Fisher's LSD test: ^∗^ *p* < 0.05  and  ^∗∗^ *p* < 0.01 are statistically significant. (b) Depletion of IL6, PAI1, and CXCL1 in an obese patient ASC CM was performed by treating with neutralizing antibodies. T24 migration was assessed in a Transwell system, and migrated cells were stained and counted. One-way ANOVA: ^∗^ *p* < 0.05, ^∗∗^ *p* < 0.01, ^∗∗∗^ *p* < 0.001, and  ^∗∗∗∗^ *p* < 0.0001 are statistically significant.

We determined whether the selected soluble factors IL8, CCL2, CX3CL1, IL6, PAI1, and CXCL1 contributed to the FCM- and ASC CM-mediated stimulation of T24 cell migration. However, exogenous treatment with CCL2, CX3CL1, and IL8 added individually did not stimulate the migration potential of T24 cells in a Transwell migration assay (data not shown). Subsequently, T24 cells were exposed to recombinant human IL6, PAI1, or CXCL1 individually placed in the lower chamber of a Transwell system. The addition of 5 ng/ml recombinant human IL6 was able to significantly enhance T24 migration in a Transwell assay, and blocking its function using a neutralizing antibody significantly reduced the migration potential (Figure 4(a)). Treatment of T24 cells with recombinant human PAI1 (40 nM, comparable to PAI1 levels found in patient ASC CM) caused a significant increase in migration (Figure 4(b)). Addition of recombinant human CXCL1 (10 ng/ml) to SFM alone was able to significantly increase the migration of T24 cells compared to the control SFM, and addition of the neutralizing antibody (10 *µ*g/ml) to deplete the exogenous recombinant CXCL1 showed significant reduction in the migratory potential (Figure 4(c)). We then tested whether blocking the function of the selected factors IL6, PAI1, and CXCL1 using a neutralizing antibody or treatment with the appropriate isotype-matched control was able to affect T24 cell migration. Interestingly, exposure of T24 cells to recombinant IL-6, PAI-1, and CXCL1 added together in the lower chamber of the Transwell system followed by the individual addition of neutralizing antibodies to either IL6, PAI1, or CXCL1 in the lower chamber significantly abrogated the ability of the ASC CM to induce T24 migration (Figure 5(a)). In addition, neutralization of IL6, PAI1, or CXCL1 together or individually in the obese ASC CM abrogated the ASC CM-mediated stimulatory effect on T24 cell migration, indicating that each of the secreted factors contributed significantly to enhance T24 cell migration *in vitro* (Figure 5(b)).

## 4. Discussion

An increasing number of studies point towards a positive association between BMI and bladder cancer risk. Therefore, it is important to understand how adipose tissue exerts its effects on the bladder tumor progression. Global gene expression profiling of adipose tissue surrounding the bladder has been applied to identify genes and regulatory pathways associated with the obese/overweight state in the context of bladder cancer. The relevant biological processes found to be altered in the obese/overweight state included the pathways of secretion and inflammation-mediated cytokine signaling.

This study demonstrates that visceral adipose tissue is a potential source of secreted factors that can induce bladder cancer cell migration *in vitro,* and the ASC population is a source of factors that have the potential to impact bladder carcinogenesis. Although whole adipose tissue explants produce a surplus of cytokines in varying quantities, we focused on IL6, IL8, CXCL1, CX3CL1, CCL2, and PAI1, whose levels were found to be secreted in high levels by the whole fat. There was no site-specific or BMI-specific trend in cytokine profiles in the whole fat explant CM. Although it was not determined if there was bladder tumor cell contamination in the whole fat specimens, the subsequent analyses utilized a population of purified and characterized ASCs that exhibited the expression of cell surface markers and adipogenic differentiation potential consistent with ASCs and not bladder tumor cells. Similar to the whole adipose tissue, high levels of IL6, IL8, CXCL1, CX3CL1, CCL2, and PAI1 were present in ASC conditioned media with no site-specific or BMI-specific trend in cytokine profiles.

Exogenous treatment of T24 bladder cancer cells using recombinant human IL6, PAI1, or CXCL1 enhanced their migratory potential. Functional blocking of these three factors using neutralizing antibodies abrogated the enhanced T24 cell migration. Neutralizing the function of IL6, PAI1, and CXCL1 in an obese patient ASC CM abrogated the ASC CM-mediated stimulatory effect on T24 cell migration. We observed that blocking of IL6, PAI1, or CXCL1 alone in the obese ASC CM abrogated the ASC CM-mediated stimulatory effect on T24 cells, indicating that each of the secreted factors contributes significantly to induce cell migration. These data provide evidence that IL6, PAI1, and CXCL1 are important factors in promoting T24 cell migration.

IL6 was previously found to be secreted by ASCs, and it caused significant stimulation of MCF-7 breast cancer cell migration, while depletion of IL6 in the ASC conditioned media decreased migration and invasion [18]. CXCL1 is reported to be produced in human breast cancer cell line MDA-MB-23 supernatants, where its downregulation is involved in the protein kinase Syk-mediated suppression of cell migration [28]. Studies in other cancers show that CXCL1 expression plays a role in regulating the invasive potential of colon carcinoma cells [29]. PAI1 is known to regulate urokinase-type plasminogen activator (uPA), by blocking its interaction with substrates. PAI1 controls local proteolysis in the extracellular matrix by inhibiting uPA [30]. However, the role of PAI1 in tumor carcinogenesis is unclear. The expression of PAI1 has been found to be elevated in tissues from patients with transitional cell carcinoma of the bladder compared to healthy controls [31]. Previous studies also report that the inhibition of PAI1 expression in T24 bladder cancer xenografts resulted in inhibition of angiogenesis and apoptosis leading to reduced tumor growth [32]. It appears that CXCL1 and PAI1 secreted by adipose tissue and bladder-derived ASCs play an important role in inducing the migration of bladder cancer cells. Targeting the cytokines secreted by ASCs in the stromal compartment and the bladder cancer cells might prove to be a new effective approach for bladder cancer therapy.

## Supplementary Materials

Supplementary Materials
*Supplementary 1.* Table 1: patient demographics.
