# Supplementary material for: Adipose Tissue-Secreted Factors Alter Bladder Cancer Cell Migration
Source: J Obes. 2018 May 20;2018:9247864. doi: 10.1155/2018/9247864 (PMC5985104; doi:10.1155/2018/9247864)
Supplement: Supplementary Materials — Supplementary 1. Table 1: patient demographics. [file 9247864.f1.docx]

Table 1: Patient Demographics

| **Patient ID** | **Age** | **Ethnicity** | **BMI** | **BMI Status** | **Tumor Stage** | **Smoking** | **Chemo**  **-therapy** | **BCG therapy** |
| --- | --- | --- | --- | --- | --- | --- | --- | --- |
| 2 | 57 | Hispanic White | 27.95 | overweight | T3 | Yes | Yes | Yes |
| 3 | 70 | Hispanic White | 33.15 | obese | T2 | Yes | No | No |
| 4 | 76 | Non-Hispanic White | 20.40 | normal | Not available | No | No | No |
| 6 | 76 | Non-Hispanic White | 31.90 | obese | T2 | Yes | No | No |
| 7 | 79 | Non-Hispanic White | 24.01 | normal | Not available | Yes | No | No |
| 8 | 70 | Non-Hispanic White | 25.07 | overweight | T2 | Yes | No | Yes |
| 10 | 75 | Hispanic White | 26.83 | overweight | T2 | Yes | No | Yes |
| 12 | 67 | Non-Hispanic White | 29.83 | overweight | T2 | No | No | No |
| 13 | 58 | Non-Hispanic White | 29.97 | overweight | T4a | Yes | No | No |
| 16 | 80 | Non-Hispanic White | 31.68 | obese | T2 | Yes | No | No |
| 17 | 56 | Non-Hispanic White | 29.81 | overweight | T0 | Yes | Yes | No |
| 21 | 60 | Non-Hispanic White | 30.66 | obese | Not available | Yes | No | No |
| 22 | 84 | Non-Hispanic White | 30.08 | obese | T1 | Yes | No | Yes |
| 23 | 51 | Hispanic White | 39.26 | obese | Not available | No | No | Yes |
| 24 | 62 | Non-Hispanic White | 41.91 | obese | T2 | No | No | Yes |
| 25 | 69 | Non-Hispanic White | 29.93 | overweight | T2 | Yes | Yes | No |
| 26 | 67 | Non-Hispanic White | 27.80 | overweight | T2 | No | No | No |
| 27 | 65 | Non-Hispanic White | 24.86 | normal | T2b | No | Yes | No |
| 28 | 72 | Non-Hispanic White | 25.85 | overweight | T2a | No | No | No |
| 30 | 64 | Non-Hispanic White | 19.21 | normal | T2 | Yes | Yes | No |
| 31 | 56 | Non-Hispanic White | 27.42 | overweight | T2 | Yes | Yes | No |
| 32 | 84 | Non-Hispanic White | 39.35 | obese | T1 | Former | No | No |
| 33 | 68 | Non-Hispanic White | 29.00 | overweight | T1 | Former | Yes | No |
| 34 | 68 | Non-Hispanic White | 33.72 | obese | T1 | Former | No | Yes |
| 35 | 75 | Non-Hispanic White | 22.18 | normal | T1 | No | Yes | No |
| 36 | 75 | Non-Hispanic White | 32.36 | obese | Ta/Tis | Former | No | Yes |
